# Circuit‐Selective FAAH Inhibition Suppresses Experimental Absence Seizures

**DOI:** 10.1002/cns.70874

**Published:** 2026-04-13

**Authors:** Tatiana P. Morais, Cristiano Bombardi, Vincenzo Crunelli, Giuseppe Di Giovanni

**Affiliations:** ^1^ Neuroscience Division, School of Biosciences Cardiff University Cardiff UK; ^2^ Department of Physiology and Biochemistry, Faculty of Medicine and Surgery University of Malta Msida Malta; ^3^ Department of Veterinary Medical Sciences University of Bologna Bologna Italy; ^4^ Institute of Pharmacology and Neurosciences, Faculty of Medicine, University of Lisbon Lisbon Portugal; ^5^ College of Medicine, Korea University Seoul South Korea; ^6^ Department of Medical and Surgical Sciences University of Magna Graecia Catanzaro Italy

**Keywords:** absence epilepsy, CB1 receptor, endocannabinoids, FAAH inhibition, GAERS, in vivo electrophysiology, PF‐04457845, spike–wave discharges, thalamocortical network, ventrobasal thalamus

## Abstract

**Background:**

Childhood absence epilepsy (CAE) arises from dysfunctional corticothalamic networks generating spike wave discharges (SWDs) and behavioral arrest. Despite available treatments, a significant proportion of patients remain pharmacoresistant and develop neuropsychiatric comorbidities. The endocannabinoid system (ECS), through activity‐dependent signaling, is a key regulator of synaptic and network stability, but its therapeutic potential in absence epilepsy remains unresolved.

**Aims:**

To determine whether selective elevation of endogenous cannabinoid tone—particularly anandamide (AEA)—via inhibition of fatty acid amide hydrolase (FAAH) suppresses absence seizures and to define the contribution of thalamic mechanisms.

**Materials and Methods:**

Video‐EEG recordings were performed in Genetic Absence Epilepsy Rats from Strasbourg (GAERS), combining automated detection and blinded validation of SWDs. The irreversible FAAH inhibitor PF‐04457845 was administered acutely and subchronically and also delivered via bilateral microinfusion into the ventrobasal (VB) thalamus. Seizure number, total seizure time, and seizure duration were quantified.

**Results:**

FAAH inhibition produced a robust and sustained reduction in absence seizures, primarily by decreasing seizure number and cumulative seizure time, with minimal effects on seizure duration. These effects were observed following both acute and repeated systemic administrations, without evidence of tolerance. Importantly, focal VB microinfusion of PF‐04457845 reproduced the anti‐absence effects, demonstrating that thalamic enhancement of endocannabinoid signaling is sufficient to attenuate pathological network activity. These effects are consistent with increased brain AEA levels and enhanced activity‐dependent CB1 receptor signaling.

**Discussion:**

Our findings indicate that selective amplification of endogenous cannabinoid signaling—likely driven by increased AEA availability—suppresses absence‐like activity by modulating thalamocortical network dynamics. In contrast to direct CB1 receptor agonists, which exacerbate absence seizures, FAAH inhibition preserves the spatial and temporal specificity of ECS, enabling circuit‐restricted modulation of excitability. The VB thalamus emerges as a critical locus for ECS‐mediated control of seizure generation.

**Conclusion:**

FAAH inhibition represents a mechanistically distinct and circuit‐selective strategy to suppress absence seizures, likely through elevation of endogenous AEA and targeted modulation of thalamocortical networks. These findings support further translational development of FAAH inhibitors as potential therapies for CAE.

## Graphical abstract

Selective inhibition of FAAH with PF‐04457845 enhances endogenous AEA signaling and suppresses absence seizures in Genetic Absence Epilepsy Rats from Strasbourg (GAERS). Video‐EEG recordings demonstrate a marked reduction in SWDs following both acute and subchronic FAAH inhibitions. These anti‐absence effects are recapitulated by focal microinjection into the VB thalamus, identifying this region as a key site of action. Mechanistically, FAAH inhibition enhances endocannabinoid (AEA) tone within thalamocortical circuits, thereby reducing pathological network oscillations underlying absence seizures.

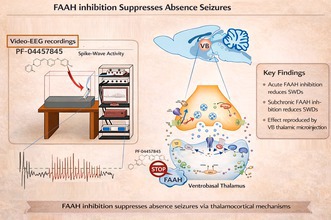

1

Childhood absence epilepsy (CAE) is characterized by frequent seizures consisting of spike–wave discharges (SWDs) and motor arrest that arise from dysfunctional cortico‐thalamic networks [1]. Although CAE has long been considered a relatively benign epilepsy, up to one third of patients are pharmaco‐resistant and many develop long‐lasting neuropsychiatric comorbidities, including attentional and memory deficits, anxiety, and mood disorders [1]. These limitations underscore the need for mechanism‐based therapeutic strategies targeting network dysfunction rather than seizure suppression alone.

Among emerging targets, the endocannabinoid system (ECS) has attracted increasing attention because of its central role in regulating neuronal excitability, synaptic transmission, and activity‐dependent plasticity [2, 3, 4]. Endocannabinoids (eCBs) act as retrograde messengers that constrain excessive neurotransmitter release and stabilize network activity. Despite the clinical success of cannabidiol (CBD) in Dravet and Lennox–Gastaut syndromes [5], evidence supporting cannabinoid‐based therapies in CAE remains limited and conflicting [3]. Notably, direct activation of cannabinoid type‐1 receptors (CB1Rs) using Δ9‐tetrahydrocannabinol or synthetic agonists consistently exacerbates absence seizures (ASs) in genetic absence epilepsy models, including WAG/Rij and GAERS rats (see [3] and reference therein).

In contrast, a growing body of preclinical evidence indicates that circuit‐restricted enhancement of endogenous cannabinoid signaling suppresses absence seizures. Focal administration of anandamide (AEA), palmitoylethanolamide (PEA), or CB1R agonists into thalamic or cortical nodes of the SWD‐generating network reduces seizure activity in WAG/Rij rats [6]. Similarly, CB1R positive allosteric modulators attenuate ASs in GAERS without producing the pro‐absence effects associated with orthosteric agonists (see [3] and reference therein). These observations suggest that the outcome of ECS modulation critically depends on both anatomical localization and mode of receptor engagement.

Here, we provide electrophysiological in vivo evidence that selective enhancement of endogenous cannabinoid tone via inhibition of fatty acid amide hydrolase (FAAH) robustly suppresses absence seizures in GAERS rats. All procedures were approved by the Cardiff University Animal Ethics Committee and conducted in accordance with ARRIVE guidelines, the Basel Declaration (3Rs), and UK Home Office regulations. Using video‐EEG recordings combined with automated detection and blinded manual validation of SWDs, we assessed the effects of the irreversible FAAH inhibitor PF‐04457845 [7] following acute (Figure 1A–G) and subchronic (7 days) systemic administration (Figure 1H–N), as well as focal bilateral microinfusion into the ventrobasal (VB) thalamus (Figure 2A–G). Across all experimental paradigms, FAAH inhibition consistently reduced ASs, primarily by decreasing seizure number and total time spent in seizures, while exerting minimal effects on mean seizure duration (Figures 1, 2).

**FIGURE 1:**
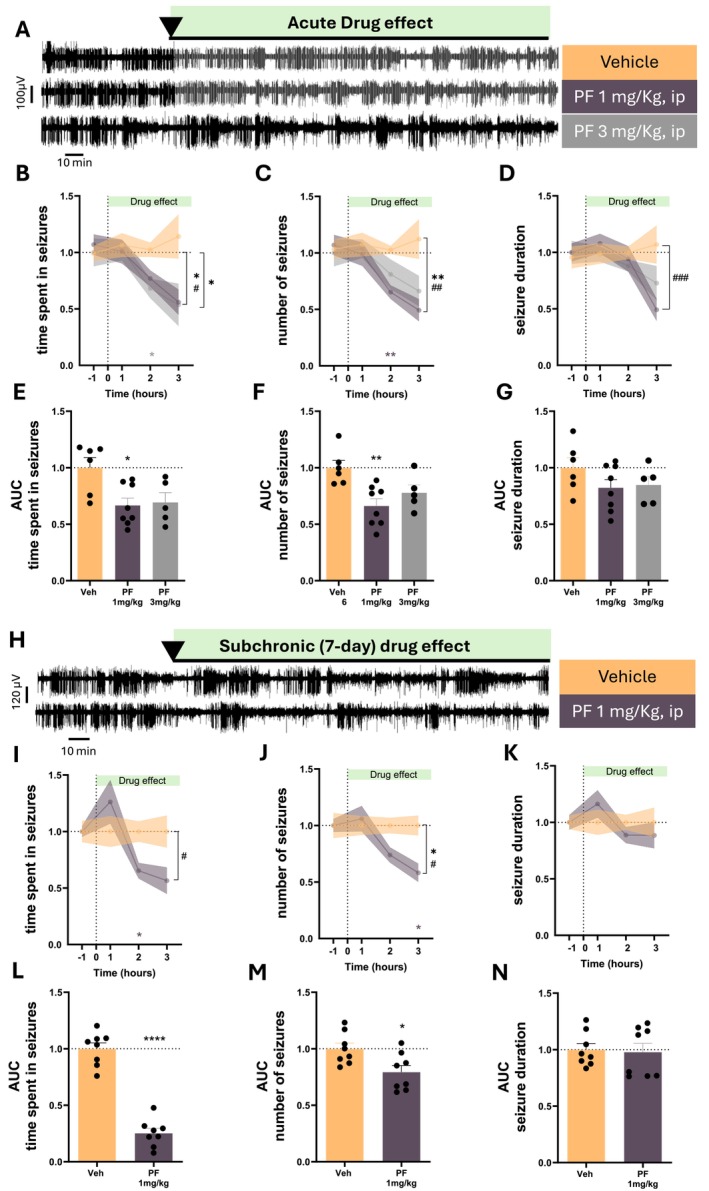
Acute and subchronic systemic FAAH inhibition suppresses absence seizures in GAERS rats. (A) Representative EEG recordings from adult GAERS rats injected with vehicle (*n* = 6), PF‐04457845 (1 mg/kg, i.p.) (*n* = 8) or PF‐04457845 (3 mg/kg, i.p.) (*n* = 5) at the indicated time. (B–D) Time course of drug effects on (B) total time spent in seizures, (C) number of seizures, and (D) mean seizure duration during baseline (−1 h) and 3 h post‐injection. PF‐04457845 reduced seizure burden at both doses. At 1 mg/kg, two‐way ANOVA revealed: Time in seizures, Treatment effect F_(1,12)_ = 4.985, *p* = 0.045 and Time×Treatment F_(3,36)_ = 4.202, *p* = 0.0119; seizure number, Treatment F_(1,12)_ = 5.346, *p* = 0.007 and Time×Treatment F_(3,36)_ = 5.093, *p* = 0.004; seizure duration, Time×Treatment F_(3,36)_ = 7.567, *p* = 0.0005. At 3 mg/kg, PF‐04457845 produced a treatment effect on time spent in seizures (F_(1,9)_ = 5.194, *p* = 0.048), with post hoc reduction at 1–2 h (*p* = 0.032). (E–G) Area under the curve (AUC) analysis across the recording period. PF‐04457845 (1 mg/kg) reduced total time spent in seizures (*p* = 0.016) and seizure number (*p* = 0.004), with no effect on seizure duration AUC. (H) Representative EEG recordings from adult GAERS rats injected with repeated PF‐04457845 (1 mg/kg for 7 days) (*n* = 8) and vehicle (*n* = 8) treatment. (I–K). Time in seizures: Time×Treatment F_(3,42)_ = 4.053, *p* = 0.012. Seizure number: Treatment F_(1,14)_ = 5.580, *p* = 0.033 and Time×Treatment F_(3,42)_ = 3.709, *p* = 0.018. Seizure duration was not significantly affected. (L–N) AUC analysis after repeated treatment confirmed reductions in total time spent in seizures (*p* < 0.0001) and seizure number (*p* = 0.017), with no effect on seizure duration. Each data point is the mean ± SEM of the cumulative results during the pre‐injection control period (−1 h) and the three hours following PF injection. Two‐way ANOVA with Sidak post hoc test was used to calculate differences between various experimental conditions for treatment (*) and Time×Treatment (#) effect and at the same timepoint (*p < 0.05, **p < 0.01, ***p < 0.001) (asterisks show post hoc test significance and asterisks within brackets show the ANOVA test significance). For the AUC analysis, One‐way ANOVA with Dunnet post hoc test was used to calculate differences between various experimental conditions (*p < 0.05, **p < 0.01).

**FIGURE 2:**
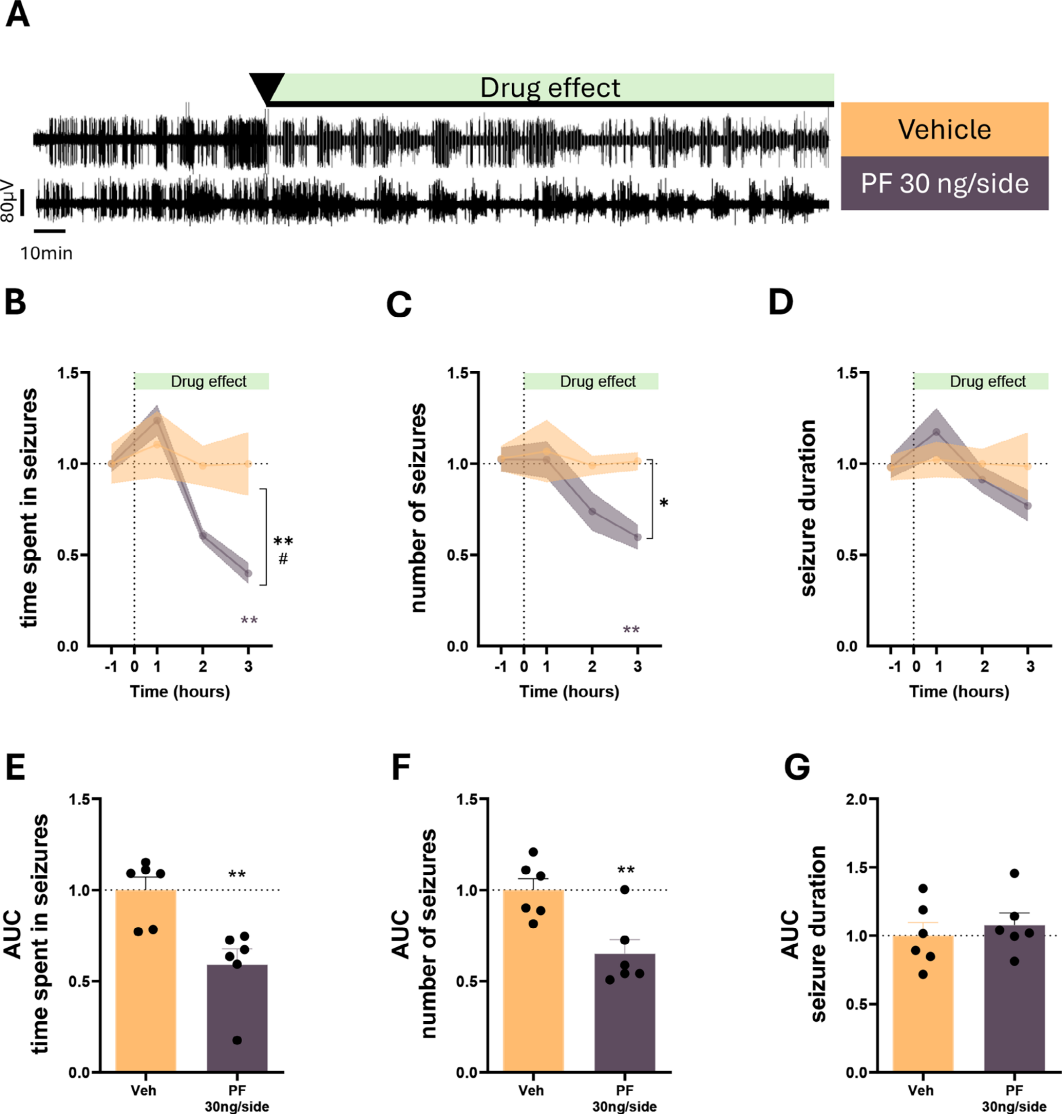
Ventrobasal thalamic FAAH inhibition is sufficient to suppress absence seizures. (A) Representative EEG traces following bilateral vehicle (*n* = 6) or PF‐04457845 infusion (30 ng/side) (*n* = 6) into the VB. (B–D) Time course of effects on seizure parameters. PF‐04457845 reduced seizure burden: Time spent in seizures, Treatment F_(1,10)_ = 11.58, *p* = 0.006 and Time×Treatment F_(3,30)_ = 4.072, *p* = 0.015; seizure number, Treatment F_(1,10)_ = 6.481, *p* = 0.029. Reductions occurred during the 1–3 h post‐infusion interval. Seizure duration was not significantly affected. (E–G) AUC analysis confirmed reductions in total seizure time (*p* = 0.004) and seizure number (*p* = 0.005), with no effect on seizure duration. Data are mean ± SEM. Two‐way ANOVA with Sidak post hoc tests; one‐way ANOVA with Dunnett post hoc tests. *p < 0.05, **p < 0.01, vs. vehicle.

Acute intraperitoneal administration of PF‐04457845 (1–3 mg/kg, i.p.) produced a clear, time‐dependent reduction in SWDs, evident within 1–3 h after injection (Figure 1A–G). Quantitative EEG analyses demonstrated a significant decrease in both seizure frequency and cumulative seizure time compared with vehicle‐treated controls. Notably, the magnitude of the anti‐absence effect was comparable at both doses, suggesting that near‐complete FAAH inhibition may already be achieved at the lower dose, consistent with the high potency and irreversible mechanism of PF‐04457845 [7]. At higher doses, compensatory mechanisms such as CB1R desensitization or synaptic saturation [4] within thalamocortical circuits may further limit efficacy.

Repeated systemic administration of PF‐04457845 (1 mg/kg, i.p. for 7 days) resulted in a more pronounced suppression of ASs, with no evidence of tolerance (Figure 1H–N). This subchronic treatment significantly reduced seizure number and total seizure time across the recording period, again without consistently affecting seizure duration. These findings indicate that FAAH inhibition produces sustained anti‐absence efficacy and may be compatible with chronic therapeutic use, an important consideration for CAE.

To determine whether thalamic FAAH inhibition is sufficient to suppress ASs, PF‐04457845 was microinfused bilaterally into the VB. Focal VB administration markedly reduced seizure number and total seizure time (Figure 2A–G), closely mirroring the effects observed after systemic treatment. This result demonstrates that enhancement of endogenous cannabinoid signaling within the VB alone is sufficient to attenuate pathological thalamocortical oscillations underlying ASs. Given the central role of VB thalamocortical neurons in the generation of pASs [1], these data highlight the VB as a critical site for ECS‐mediated seizure control.

The present findings are mechanistically relevant in view of previous evidence indicating altered ECS signaling in experimental ASs. GAERS and WAG/Rij rats display region‐specific changes in CB1R expression and endocannabinoid levels within cortico‐thalamic circuits [8]. Importantly, FAAH inhibition enhances on‐demand AEA signaling while preserving the spatial and temporal specificity of endogenous eCB release, thereby avoiding the widespread receptor activation produced by direct CB1R agonists. This distinction likely explains why FAAH inhibition suppresses ASs, whereas orthosteric agonists exacerbate them.

From a translational perspective, PF‐04457845 has been extensively evaluated in humans and shown to be safe and well tolerated, producing near‐complete FAAH inhibition and robust elevation of AEA levels without major adverse effects [9]. Although clinical trials did not demonstrate efficacy for chronic pain [10], PF‐04457845 reduced cannabis withdrawal symptoms and cannabis use [9], supporting its CNS penetration and functional engagement of FAAH inhibition in humans. Importantly, the absence of overt psychoactive effects [3] distinguishes FAAH inhibition from direct cannabinoid receptor agonism and represents a major advantage for potential use in pediatric epilepsies such as CAE.

Beyond its immediate translational relevance, FAAH inhibition also provides a valuable experimental tool to dissect the contribution of endogenous cannabinoid signaling to cortico‐thalamic network dynamics. By selectively amplifying activity‐dependent endocannabinoid release, FAAH inhibition allows endogenous circuits to retain control over the spatial and temporal domain of cannabinoid signaling. This property is particularly relevant in absence epilepsy, where pathological oscillations emerge from highly structured cortico‐thalamic loops and are exquisitely sensitive to small changes in synaptic balance [1].

In conclusion, we provided EEG‐based functional evidence that circuit‐selective enhancement of endogenous cannabinoid signaling via FAAH inhibition suppresses ASs in GAERS rats. By preferentially reducing seizure initiation without altering seizure duration, FAAH inhibition offers a mechanistically distinct approach compared with existing therapies and reconciles previously conflicting results of cannabinoids' effects on ASs. These results support further preclinical and clinical evaluation of FAAH inhibitors as potential treatments for CAE.

## Author Contributions

**Tatiana P. Morais:** investigation, methodology, data curation, formal analysis, visualization, writing – original draft, writing – review and editing. **Cristiano Bombardi:** writing – review and editing. **Vincenzo Crunelli:** conceptualization, supervision, writing – review and editing, funding acquisition. **Giuseppe Di Giovanni:** conceptualization, supervision, project administration, writing – original draft, writing – review and editing, funding acquisition.

## Funding

This work was supported by Wellcome Trust, 91882. Malta Council for Science and Technology, REP‐2020‐006.

## Conflicts of Interest

The authors declare no conflicts of interest.

## Data Availability

The data that support the findings of this study are available from the corresponding author upon reasonable request.
